# Neoliberalism 4.0: The Rise of Illiberal Capitalism: Comment on "How Neoliberalism Is Shaping the Supply of Unhealthy Commodities and What This Means for NCD Prevention"

**DOI:** 10.15171/ijhpm.2019.111

**Published:** 2019-11-11

**Authors:** Ronald Labonté

**Affiliations:** Globalization and Health Equity, Faculty of Medicine, School of Epidemiology and Public Health, University of Ottawa, Ottawa, ON, Canada.

**Keywords:** Liberalism, Neoliberalism, Capitalism, Health Inequities

## Abstract

Neoliberal logic and institutional lethargy may well explain part of the reason why governments pay little attention to how their economic and development policies negatively affect health outcomes associated with the global diffusion of unhealthy commodities. In calling attention to this the authors encourage health advocates to consider strategies other than just regulation to curb both the supply and demand for these commodities, by better understanding how neoliberal logic suffuses institutional regimes, and how it might be coopted to alternative ends. The argument is compelling as possible mid-level reform, but it omits the history of the development of neoliberalism, from its founding in liberal philosophy and ethics in the transition from feudalism to capitalism, to its hegemonic rise in global economics over the past four decades. This rise was as much due to elites (the 1% and now 0.001%) wanting to reverse the progressive compression in income and wealth distribution during the first three decades that followed World War Two. Through three phases of neoliberal policy (structural adjustment, financialization, austerity) wealth ceased trickling downwards, and spiralled upwards. Citizen discontent with stagnating or declining livelihoods became the fuel for illiberal leaders to take power in many countries, heralding a new, autocratic and nationalistic form of neoliberalism. With climate crises mounting and ecological limits rendering mid-level reform of coopting the neoliberal logic to incentivize production of healthier commodities, health advocates need to consider more profound idea of how to tame or erode (increasingly predatory) capitalism itself.

In their insightful article, Lencucha and Thow^1^ invoke new institutional theory to argue that the 40-year dominance of neoliberal logic in politics and economics has embedded itself within governments’ policy choices. The article focuses on the trinity of unhealthy commodities (tobacco, obesogenic foods, and alcohol). The authors acknowledge that one oft-studied and important determinant of the continued production and global diffusion of these commodities is the influencing power of private economic actors on governments’ decision-making. They posit, however, that a less obvious determinant is how neoliberal ideas have permeated governmental and global governance institutions, reconfiguring the role of the state in mediating between markets and civil society. They conclude that a better understanding of how neoliberal assumptions become taken-for-granted, particularly in the development policies of low- and middle-income countries, provides new avenues for how health actors might engage to reduce the production and diffusion of unhealthy commodities, beyond simply the call for stronger forms of public regulation.

There is nothing to fault with the main contours of their argument. It is one that I have also promulgated for several years, assessing the health harms that flow from policies based on neoliberal theory: from neoliberalism 1.0 (the structural adjustment programs of the 1980s and 1990s) to neoliberalism 2.0 (the financialization of the global economy made possible by technology and de-regulation) to neoliberalism 3.0 (fiscal austerity in the wake of government bailouts of the 2007/2008 financial crisis and ensuing recession).^2,3^ As my title for this commentary nonetheless suggests, it is important to view the challenges facing health equity advocates in light of new political dynamics re-shaping once more the pathological practices of our (still globalized but increasingly fractious) capitalist economies. To that end, my commentary is an effort to extend, rather than to critique, the analysis presented by the authors.

## From Liberalism to Neoliberalism

Neoliberalism’s core values of “individual liberty and freedom” highlighted by the authors are not exclusive to its past 40 years of ideological dominance, but date back at least to the rise of Western philosophical liberalism during the transition from feudalism to capitalism. Responding to rationalism’s dethroning of religious beliefs and democracy’s displacement of the divine right of kings, writers such as John Locke and Adam Smith propounded on the importance of individual rights to free speech, freedom of religion, free markets, and ownership of property. These were radical ideas at the time, helping to foment revolutions in England, the United States, France, and across Europe, even if such rights tended to entitle primarily the white, male, bourgeois class.

More recently, one can find these same norms embedded in international human rights treaties, which nominally are agnostic as to economic or political systems and their respective institutions and are not usually associated with neoliberalism as a political philosophy or movement. Moreover, human rights treaties in practice straddle state obligations to protect individual rights to ‘liberty and freedom’ on the one hand, while ensuring (progressively) provision of certain core services thought essential to human flourishing.^4^ This differs little from the ambivalence found in Adam Smith’s own writings. Neoliberals invoke his economic arguments on manufacturing efficiency and the market’s ‘invisible hand’ transforming the rational pursuit of individual gain into collective betterment^ [1] ^, but ignore his moral writings on the subsequent inequality and impoverishment this could create and the importance of the state to do something about it. The social contract between state and civil society has long been challenged by the fiscal contract between state and market, with neoliberalism, in many ways, simply laissez-faire liberal capitalism on steroids.^5^ Similarly, neoliberalism is not alone in extolling “the generic rationality of economic growth” (p. 2) as the authors note, since economic growth has long been a dominant, if not *the* dominant, feature of capitalism, just as imperial expansion was always a feature of previous eras of empires and feudal states.

## Neoliberalism’s Rapid Ascent

Much has been written about von Hayek, the progenitor of modern ‘neo’-liberalism, and his concerns with the rise of communism and the post-war popularity of social democracy, which he thought would lead to totalitarianism and put Western liberalism back on a *The Road to Serfdom* , as his famous 1944 book was titled.^6^ His prescriptions for an extreme liberalism dismissed the role of governments in managing the economy, in contrast with German ordo-liberalism, a contemporaneous theory embracing free markets but claiming the need for their strong government management to avoid a degeneration into fascism.^7^ Both ideas of a liberalism redux, however, were eclipsed by ‘les trente glorieuses,’ the French phrase for the three decades following World War Two characterized by rapid economic growth, improved wages and living conditions, new forms of government social protection measures, and new progressive social movements.^8^ Keynesian economics prevailed during this period, emphasizing *inter alia* the importance of government demand management, countercyclical spending in times of recession, and redistributive tax and transfer programs to buffer market inequalities. The ‘developing’ (and de-colonizing) world, in turn, largely embraced import substitution as the model of economic growth, deployed the bipolar (US/USSR) political world to advantage whenever possible, and called for creation of a ‘new international economic order’ predicated, in part, on reparation for the injuries suffered during the colonial era.^2^

This ‘golden era,’ as others have described the three post-war decades,^9^ quickly unraveled when the United States abandoned the gold standard leading to financial arbitrage. A few years later oil cartel price shocks created economic recessions and monetarist efforts (the sudden sharp rise in interest rates) to jolt the rich world out of stagflation risked developing countries’ defaulting on loans from rich world banks. These geopolitical developments, as Lencucha and Thow correctly recount, gave neoliberalism’s playbook its opening. Carefully nurtured over the decades by von Hayek, Friedman, Popper, and other acolytes of the Mont Pelerin Society, the neoliberal playbook fundamentally opposes “the expansion of government, not least in state welfare [and] the power of trade unions and business monopoly,”^10^ all regarded as imminent dangers to “Western civilization” evident in the threat of creeping socialism. Its policy nostrums have so far proven ineffective in preventing monopolies, but have been extremely adept in privatizing government, restraining state welfare, crippling trade unionism, and creating the seemingly unstoppable wealth ascendency of the 0.001%.^2^ The Mont Pelerin agenda and its subsequent impacts lays bare the ideological and political nature of neoliberalism, well distant from its original base in a moralizing and (putatively) democratizing classical liberalism.

In their emphasis on the role that policy legacies play in perpetuating neoliberal tenets, Lencucha and Thow, perhaps necessarily, omit reference to the important ‘why’ and ‘how’ neoliberalism came to so successfully colonize the recessionary niche of the late 1970s and early 1980s. Political economists, such as Harvey^11^ and Wade,^12^ explain neoliberalism’s ascendency by underscoring the centrality of politics—the election of conservative governments in the United States (Reagan) and the United Kingdom (Thatcher) in the 1980s—and powerful influence-wielding individuals: economic elites who had seen their unfair share of pre-War wealth erode into a more equitable distribution during the post-War Keynesian decades. As Wade summarized:

“The hidden agenda of the Reagan/Thatcher revolution was to reverse this ‘Great Compression’ and allow income and wealth to be restored to their rightful owners at the top—combining market liberalisation with an array of state measures which had the effect, intended and unintended, of intensifying redistribution upwards.”^13^

Yes, neoliberal ideas sedimented in government development policies and economic ministries through their diffusion by powerful global institutions and a cohort of neoliberally trained economists, as Lencucha and Thow point out. But these ideas remain cemented in place more by their usefulness to global elites and predatory (increasingly rentier) capital accumulation than by any policy path dependency per se. To emphasize the one without the other leaves a blind spot for health advocacy engagement with the new ‘illiberalism’ of neoliberal capitalism that we are now experiencing by failing to ask: who benefits?

## Confronting an Illiberal Future

The 2016 election of Donald Trump is regarded by many as the beginning of the end of neoliberal globalization’s four decades of hegemonic dominance^ [2] ^. At the very least, many of its underlying economic tenets (notably the legal/institutional rules governing global trade) are under threat of the protectionism and trade wars that were dangerous preludes to past world wars which liberalization’s deep market integration was intended to prevent. There are undoubtedly strong echoes of these neoliberal tenets within the ‘institutional configurations’ that are the authors’ focus, and one that I have shared with them in past and ongoing research. These echoes condition and constrain the policy options from which many governments might choose and, on one level, Lencucha and Thow are correct to conclude that these residual tenets could be invoked to incentivize the supply side of healthier commodities while protecting farmer livelihoods. Over time, success in doing so could reduce demand for a diminishing supply of unhealthy products. On another level, however, this reasoning rests on neoliberalism’s underlying logic of continuous economic growth which the authors earlier criticize for being dislocated from social policy concerns, to say nothing of our Extinction Rebellion-era need to address the ecological limits of growth.

Moreover, the older neoliberal rules of the game are in flux, consequent to their unparalleled success in allowing a small number of global elites to create a new gilded age for themselves by confining the vast majority of humankind to a (slightly less extreme) poverty, or to a declining share of wealth and a sluggish economy propped up by unprecedented levels of debt. Global governing institutions of neoliberalism 1.0 through 3.0 (the World Bank, the International Monetary Fund, even the World Trade Organization first constructed under US aegis but which the Trump administration seems keen to undermine by rendering its appellate body ineffectual) are struggling to find their place in a new multipolar world. This leads some to declare the demise of globalization, at least we have recently known it.^13^ Whether this portends to a new world war, a series of regional wars, or more democratic global governing systems remains grist for speculation, although the present is decidedly marked by an increase in undemocratic and autocratic regimes. As Roberto Savio, the Italian/Argentinian journalist, recently noted:

*“…the right has managed to capitalize on the broad and growing hostility to globalization, rooted especially in the feeling of being left behind experienced by working-class people. Prior to the US financial crisis of 2008 and the European sovereign bond crisis of 2009, the National Front in France was the only established right-wing party in the West. Since then, with a decade of economic chaos and brutal austerity, right-wing parties have blossomed everywhere.”* ^14^

Does this mean the end of either globalization or its neoliberal agenda, as some suggest?^15^ Probably not. In his brilliant excoriation of neoliberalism’s disequalizing inevitability, the heterodox development economist, Jose Gabriel Palma, notes that “the current re-emergence of neofascism” (a less polite way to describe our autocratic and increasingly xenophobic body politic) is marked by its “tendency to mix extreme-right politics and ‘dark ages’ morality, with exactly the same primordial neoliberal economics and acute inequality.”^16^ There may still be a push for open markets, but only if unabashedly benefiting the geopolitical interests of more powerful states and the economic interests of their elites – a world in which bilateral power trumps any pretext of multilateral equity. The ‘real economy’ of production and consumption (whether of healthy or unhealthy commodities) will persist but will play second fiddle to a global financialized economy that thrives and grows despite 2008’s global financial meltdown. Governments in many countries will continue to exercise fiscal austerity except in areas benefiting their military or economic power. Global tax competition and national tax policies are more likely to retain their upwards, rather than downwards, redistribution.

‘Primordial neoliberalism’ is little other than a return to the foundations of mercantilist capitalism itself, the difference now being that democratic politics is stagnating, if not declining. Neoliberalism 4.0, our unfolding era of illiberal capitalism, or what others have called “authoritarian neoliberalism,”^17^ represents a new iteration of an old maxim that, as the Financial Times economics commentator, Martin Wolf, wrote in 2016, “Democracy is egalitarian. Capitalism is inegalitarian.”^18^ A terminal collision is inevitable.

My comments do not invalidate the arguments made by Lencucha and Thow, which are mid-level spot-on. But they do add a new and increasingly urgent macro-level agenda for health advocates: the taming or complete erosion of capitalism, a system that has outgrown its emancipatory promise and, in its most recent illiberal iteration, now threatens the very bases of human life. There are policy roadmaps for its taming (albeit rarely implemented) but only hints (circular economies, cooperatives) of its more thorough transformation.

## Ethical issues

Not applicable.

## Competing interests

Author declares that he has no competing interests.

## Author’s contribution

RL is the single author of the paper.

## Endnotes


[1] Although it is worth noting that Smith ignored how the ‘wealth’ this benign market created in (some) ‘nations’ involved the gross exploitation of peoples and lands in others.



[2] See for example: Fraser N, The End of Progressive Neoliberalism, Dissent January 2, 2017 (http://www.bresserpereira.org.br/terceiros/2017/fevereiro/17.02-End-of-Progressive-Neoliberalism.pdf) Accessed November 3, 2019; and Smith A, Trump and the crisis of the neoliberal world order, Int Soc Rev 105 2019 (https://isreview.org/issue/105/trump-and-crisis-neoliberal-world-order) Accessed November 3, 2019.

